# The novel mechanism of financing effect on companies development empirical study

**DOI:** 10.1371/journal.pone.0326934

**Published:** 2025-07-18

**Authors:** Siwei Tao, Xiuling Yuan

**Affiliations:** 1 China Shipbuilding Trading (Shanghai) Co., Ltd, Shanghai, China; 2 School of Management Engineering and Business, Hebei University of Engineering, Handan, China; American University of the Middle East, KUWAIT

## Abstract

As China’s economy continues to expand rapidly, the demand for capital among enterprises has surged, leading to an increased preference for short-term loans intended for long-term investments. An analysis of relevant data reveals that utilizing short-term financing for long-term projects positively influences the leverage of non-financial listed companies. Notably, private enterprises experience greater benefits compared to their state-owned counterparts. While short-term loans effectively enhance short-term leverage, their impact on long-term leverage appears to be minimal. Consequently, it is advisable for businesses to select financing strategies that align with their specific circumstances and to enhance their financial management practices to create new funding avenues. Additionally, there is a call for the government to bolster support for private enterprises. These findings hold significant implications for guiding financial decision-making and management in enterprises.

## 1. Introduction

In the current economic situation, small and medium-sized enterprises (SMEs) are suffering from the severe challenge of tight financial chains, which greatly limits their growth and competitiveness [1]. Especially in the context of increased economic volatility and rising market uncertainty, the problems of inefficient and costly financing and poor financing channels are becoming more and more prominent [2]. Due to their limited size, short credit history and insufficient collateral assets, SMEs often have difficulty in obtaining the necessary loan support, making the road to financing extremely difficult [3].

In order to cope with the problem of insufficient liquidity, many small and medium-sized enterprises have turned to the financing mode of “short-term borrowing, long-term investment” [4]. The basic idea of this model is to use short-term loans to raise funds quickly, and then invest in long-term projects in order to obtain a higher return on investment [5]. Although this approach can alleviate the financial pressure in the short term, its potential risks should not be ignored [6]. Long-term investment return cycle is long, if it fails to achieve profitability, the enterprise may fall into the predicament of capital chain breakage, which may lead to a deeper financial crisis. In addition, the high interest burden associated with short-term loans will also constitute a heavy pressure on the overall financial situation and future development potential of enterprises [7].

In this case, risk and return assessment of short-term loans and long-term investments becomes crucial [8]. Among them, how to assess the impact of short-term loans on the leverage ratio of enterprises has become a topic worthy of in-depth study. As an important indicator to measure the financial risk of enterprises, the change of leverage ratio is not only related to the financing cost of enterprises, but also directly affects the capital structure and investment decisions of enterprises [9]. This research explores the relationship between short-term loans and corporate leverage, aiming to identify key determinants and their operational mechanisms. Such insights are crucial for providing SMEs with effective financing strategies and risk management frameworks. While previous studies have examined various aspects of financing, including the comparison of short-term versus long-term loans on investment efficiency [10], the effects of policy uncertainty on leverage [11], and factors like enterprise size and asset turnover on leverage [12], there remains a scarcity of comprehensive analyses regarding short-term loans utilized for long-term investments in relation to leverage. This study specifically investigates how short-term borrowing influences long-term investment decisions and corporate leverage, clarifying the underlying mechanisms and factors involved through thorough data analysis and empirical research. Additionally, it aims to offer actionable recommendations and risk mitigation strategies tailored to the unique circumstances of SMEs, thereby supporting their sustainable growth and enhancing their competitive edge within the economic landscape.

## 2. Literature review and research hypothesis

### 2.1 The positive impact of short-term financing on corporate leverage

In today’s fast-changing financial markets, enterprises are faced with the dual challenge of improving competitiveness and expanding their scale [13–15]. Many enterprises rely on short-term borrowing to obtain the liquidity they need in order to meet their day-to-day operational needs. However, while short-term debt can help firms access capital quickly, its use also poses complex risks and potential negative effects.

However, short-term debt must be used cautiously because an excessive short-term debt ratio may negatively affect the firm’s return on investment [18], which in turn affects the firm’s long-term sustainability. More importantly, when firms use short-term debt for long-term investments, it may lead to inadequate disclosure and low transparency, which in turn affects firm performance and increases the risk of non-compliance [16,17]. This risk not only undermines investor trust, but also exposes firms to scrutiny from regulators and increases compliance costs.

In terms of leverage ratios, studies have shown that higher leverage ratios are usually associated with higher levels of working capital [19]. However, an increase in the leverage ratio may also negatively affect the firm’s profitability by reducing profits [20] and further affecting the overall value of the firm [21]. Therefore, enterprises should pay great attention to the leverage ratio as an important indicator of solvency and prioritize the gearing ratio, current ratio and quick ratio when making financing decisions [22]. Considering the above factors, this study proposes the following hypotheses:

***Hypothesis 1*** *: Short-term loans for long-term investments significantly increase the leverage of the corporate sector.*

In order to deeply analyze the specific role of short-term loans on leverage over different time horizons, based on Hypothesis 1, we further refine it into two sub-hypotheses, H1a and H1b. Short-term leverage directly reflects the firm’s financial flexibility and risk-taking ability in the short term, while long-term leverage maps more to the firm’s overall financial structure and its long-term profitability. Clarifying the impact mechanism of these two can enable us to more thoroughly understand the different financial effects brought by short-term loans for long-term investment, and then provide more accurate and practical guidance programs for corporate financing decisions.

*H1a: Short-term loans that are used for long-term investments will significantly increase the short-term leverage ratio.*

*H1b: Short-term loans used for long-term investments will have a relatively limited and insignificant effect on the long-term leverage ratio.*

### 2.2 The effect of short-term debt on the leverage ratio of non-state-owned firms

In China’s enterprise categorization, there are significant differences between SOEs and non-SOEs in terms of access to credit, financing advantages and political connections [23]. SOEs usually enjoy financial and political assistance from the government, which gives them a significant advantage in accessing credit [24]. Due to the natural interest relationship between SOEs and state-owned banks, these firms tend to receive lower bank performance expenses [25]. Creditworthy SOEs are more likely to repay their loans on time when financing, thus further strengthening their position in the credit market [26].

In addition, political connections play an important role in firms’ access to bank loans [27]. The more the government intervenes, the smaller the proportion of long-term debt of firms tends to be, which makes it easier for credit funds to flow to SOEs [28]. Although SOEs bear more policy burdens in their operations, they also receive more policy support as a result [29]. These policy supports are not only reflected in the intervention in the allocation of bank credit funds, but also in the reduction of financing costs for SOEs.

However, unlisted firms face greater challenges in financing. Due to the lack of strong political ties with the government and banks, non-listed firms have relatively weak bargaining power in the credit market [30]. They usually have higher financing costs and face more scrutiny and restrictions in obtaining credit. This makes it particularly risky and uncertain for non-state firms to utilize short-term loans for long-term investments [31].

State-owned enterprises are able to make better use of short-term loans for long-term investment in the financing process, and therefore face relatively few financial crises; whereas the challenges faced by non-state-owned enterprises in this process are more complex and severe [32]. The greater impact of short-term loans on the leverage of non-state enterprises is mainly due to the disadvantages of non-state enterprises in financing channels and access to resources. Based on the above analysis, we propose the following hypothesis:

***Hypothesis 2:*** *Short-term loans for long-term investments have a greater impact on the corporate leverage of non-state-owned firms compared to state-owned firms.*

## 3. Research design and variable selection

### 3.1 Sample selection and data sources

In order to ensure the standardization of statistical data, this paper selects the relevant financial data of Shenzhen and Shanghai A-share listed companies from 2013 to 2022 as the research samples, and the data comes from Guotai Junan database. During the sample screening process, financial and insurance listed companies are excluded to eliminate the impact of differences between their accounting and non-financial companies. In addition, in order to ensure the completeness of the research data, companies recognized as ST and *ST were excluded, and listed companies with insufficient financial data were eliminated to improve the accuracy of the research conclusions. At the same time, a tailing adjustment was made for continuous variables to eliminate the effect of biased beliefs. Finally, a total of 23,742 observations were obtained in this paper, and the data were mainly analyzed using Stata 17.0. These data were shown in the Supporting Information.

### 3.2 Model settings

This study presents a model designed to assess the impact of short-term loans on long-term investment behavior and corporate leverage ratios:


$${\text{Lr}}_{\text{it}}=\alpha_{0}+\partial_{1}{\text{SFLI}}_{\text{it}}+\partial_{2}{\text{GROW}}_{\text{it}}+\partial_{3}{\text{TANG}}_{\text{it}}+\partial_{4}{\text{CURR}}_{\text{it}}+\partial_{5}{\text{ROA}}_{\text{it}}+\partial_{6}{\text{ROE}}_{\text{it}}+\partial_{7}{\text{HLD}}_{\text{it}}+\varepsilon_{\text{it}}$$ (1)

In the model presented, SFLI is utilized to represent long-term investment, while Lr denotes corporate leverage. To enhance the model’s precision, additional control variables have been incorporated, including corporate growth (GROW), tangible assets ratio (TANG), current ratio (CURR), return on assets (ROA), return on equity (ROE), and the proportion of large shareholders’ ownership (HLD).

Based on the theory of financing constraints, the theory of capital structure, and the theory of firm growth, this study focuses on the obstacles that firms face in the financing process and examines the impact of short-term loans on long-term investment decisions.

In 1984, Stewart Myers and Nicholas Majluf proposed the theory of capital constraints, which emphasizes that firms tend to prioritize internal financing and thus experience bottlenecks in exogenous financing [33]. This theory measures the financing behavior through a certain quantitative index, reveals the financing choices of firms in different environments, and analyzes in depth the impact of financing constraints on firms’ investment capabilities and decision paths.

On the other hand, capital structure theory focuses on the role of a firm’s choice of debt or equity financing on its financial performance. The theory originated from Modigliani and Miller, who argued that under ideal market conditions, capital structure does not affect firm value [34]. However, the reality of market imperfections has prompted scholars to further explore the actual effect of capital structure on firm performance.

Firm growth theory, on the other hand, emphasizes the importance of investment and expansion for the sustained growth of a firm, pointing out that the growth of a firm is closely linked to its financing ability. Financing ability not only directly affects investment decisions, but also relates to a firm’s market competitiveness and long-term growth potential. In this theory, specific metrics such as SFLI are used to measure the relationship between long-term investments and short-term loans. Corporate growth (GROW) and other financial indicators also reveal the direct impact of capital structure choice and financing ability on corporate investment behavior.

In the theoretical model, we assume that there is a positive association between short-term loans and long-term investment. At the same time, in order to fully assess the financial health of enterprises, the impact of other variables (e.g., GROW, TANG) on long-term investment should also be considered.

### 3.3 Variable description

#### 3.3.1 The dependent variable.

Given that the gearing ratio serves as a widely recognized and representative measure for determining a company’s leverage, this study will utilize it to derive the ratio. The gearing ratio acts as a holistic metric for evaluating a company’s indebtedness, reflecting the relationship between total liabilities and total assets. In addition, this study seeks to investigate how short-term lending affects long-term investment within the framework of corporate leverage over two different time periods by categorizing corporate leverage into short-term and long-term components. This investigation will provide valuable insights into the impact of short-term financing on long-term investments for firms with different maturities.

#### 3.3.2 Explain variables.

Short-term loans for long-term investments (SFLI). In this paper, we use the current growth of long-term loans in the balance sheet and the related information in the cash flow statement. According to the formula “Long-term investment short-term borrowing level = cash paid for the purchase and construction of fixed assets, intangible assets and other long-term assets - (increase in long-term borrowings for the current period + increase in owner’s equity for the current period + net cash flows from operating activities + net cash received from the disposal of fixed assets, intangible assets and other long-term assets) to exclude economies of scale from the previous year’s total assets Calculate the level of short-term borrowings for long-term investments”. The formula for assessing existing long-term loans can be expressed as follows: existing long-term loans = current long-term loans + liabilities due within one year – previous long-term loans. If the outcome of this calculation is positive, it suggests a financial mismatch within the enterprise. Moreover, the greater the resulting value, the more pronounced the discrepancy between short-term loans and long-term investments in the organization.

#### 3.3.3 Control variables.

With reference to previous studies, this paper controls the variables of enterprise characteristics that may have an impact on enterprise leverage when analyzing enterprise leverage. Table 1 details the control variables used in this study and their calculation methods, which play a key role in analyzing the relationship between corporate leverage and long-term investment.

**Table 1:** Specific calculation instructions for each variable.

	Variable	Symbol	Method of calculation
Explained Variable	Corporate leverage ratio	Lr	total liabilities/total assets
	Short term leverage ratio	SIr	current liabilities/total assets
	Long term leverage ratio	LIr	non current liabilities/total assets
Explanatory variable	Short-term loan for long-term investment	SFLI	Long-term investment value = short-term loan amount/ 1 – interest rate x time
Control variable	Enterprise Growth	Growth	(Income for the current period – income for the previous period)/income for the current period
SIr	Tangible asset ratio	TANG	Tangible assets/total assets
	Current ratio	CURR	current assets/current liabilities
	Return on assets	ROA	net profit/total assets
	Return on equity	ROE	net profit/owner’s equity
	Major shareholder accounts	HLD	(other receivables – other payables)/total assets

The growth potential of an enterprise is measured by the growth rate of its operating income, i.e., it is reflected in the growth of the current period compared to the previous period. This indicator is particularly important for growth companies.

The short-term leverage ratio (SIr) and the tangible assets ratio (TANG) serve as the focus of attention. The short-term leverage ratio reveals a firm’s reliance on short-term financing by calculating the ratio of current liabilities to total assets. The tangible assets ratio, on the other hand, is calculated by total assets after deducting goodwill and intangible assets, reflecting the solidity of a firm’s asset structure. These two indicators have a direct impact on the financial stability of a firm.

The current ratio (CURR), as another important indicator of liquidity, reflects an enterprise’s ability to repay its debts in the short term through the ratio of current assets to current liabilities. Firms with higher liquidity are usually smoother in financing and more flexible in long-term investment.

For profitability, the study uses two indicators: return on assets (ROA) and return on equity (ROE).ROA is measured by the ratio of net profit to total assets, while ROE is the ratio of net profit to shareholders’ equity.

The corresponding empirical data was in Supporting Information, as was shown in Table S1. The indicator of major shareholders’ accounts (HLD) reveals the potential influence of shareholders on the financial position of the company by calculating the difference between other accounts receivable and total assets.

## 4. Empirical results and analysis

### 4.1 Descriptive statistics

The descriptive statistics of each variable are shown in Table 2. Overall, the leverage level of the sample firms is moderate, and the mean value of the firms’ leverage ratio is 0.456, indicating that the firms have maintained a certain balance in their financing structure. However, the mean value of the short-term leverage ratio is 0.312 and the standard deviation is 0.223, showing significant variability, which may reflect the differences in short-term financing strategies of the firms, and some of the firms may be more reliant on short-term financing to cope with liquidity needs. Meanwhile, the low mean value of long-term leverage ratio (0.092) and the negative mean value of short-term loans for long-term investment (−0.097) suggest that many firms are more conservative in· long-term investment, which may be affected by market environment and risk management. In addition, the lower mean values of firms’ growth potential and return on assets (0.111 and 0.031, respectively) hint at the relative disadvantage of the sample firms in the competitive market, which may limit their ability to raise finance and the sustainability of their long-term investments. The mean value of the current ratio of 2.077, on the other hand, indicates the relative robustness of the firms in liquidity management, despite the significant variability between current assets and liabilities. These statistics not only provide important background information for understanding the financial situation and financing behaviors of firms, but also reveal the challenges and opportunities that may be faced during the financing decision-making process, emphasizing the need for firms to optimize their capital structure and enhance their financial performance.

**Table 2:** Specific calculation instructions for each variable.

Variable	N	Mean	p50	SD	Min	Max
Lr	23742	0.456	0.401	0.153	0.056	0.875
SIr	23742	0.312	0.383	0.223	0.034	0.990
LIr	23742	0.092	0.056	0.081	0.000	0.430
SFLI	23742	−0.097	−0.061	0.184	−1.201	0.225
Growth	23742	0.111	0.093	0.259	−0.664	1.557
TANG	23742	0.387	0.397	0.242	−0.148	0.723
CURR	23742	2.077	1.070	1.832	0.325	9.895
ROA	23742	0.031	0.030	0.052	−0.328	0.166
ROE	23742	0.048	0.064	0.131	−1.268	0.319
HLD	23742	−0.019	−0.011	0.046	−0.207	0.118

### 4.2 Correlation analysis

The analysis of the data presented in Table 3 supports the first hypothesis, revealing a notable positive correlation between corporate leverage and short-term loans for long-term investments, after excluding other variables. The correlation coefficient between the corporate leverage ratio and short-term leverage ratio is 0.641, which is significant at the level (***) indicating a meaningful and statistically relevant relationship. This strong correlation suggests that as firms increase their leverage, there is a corresponding rise in short-term leverage, demonstrating the consistency in their financial decision-making.

**Table 3:** Specific calculation instructions for each variable.

	Lr	SIr	LIr	SFLI	Growth	TANG	CURR	ROA	ROE	HLD
Lr	1									
SIr	0.641***	1								
LIr	0.388***	0.102***	1							
SFLI	0.088***	0.151***	−0.011	1						
Growth	−0.013	−0.017***	0.004	−0.210***	1					
TANG	−0.693***	−0.842***	−0.488***	−0.038***	−0.028***	1				
CURR	−0.676***	−0.847***	−0.234***	−0.051***	−0.025***	0.654***	1			
ROA	−0.276***	−0.381***	−0.208***	−0.345***	0.198***	0.341***	0.248***	1		
ROE	−0.220***	−0.153***	−0.075***	−0.371***	0.252***	0.165***	0.077***	0.724***	1	
HLD	−0.250***	−0.173***	−0.012*	0.017**	−0.013	0.283***	0.162***	0.057***	0.052***	1

Note: In brackets are robust T-values from clustering to the household level, where *, **, **** and respectively indicate significant at the 10%, 5%, and 1% level, all of which are the same in the following tables.

Although the correlation coefficient between the short-term leverage ratio and the long-term leverage ratio is low at 0.102 (***), it still shows some positive correlation. This relationship suggests that under certain conditions, firms have some degree of interaction between short-term and long-term financing, especially in terms of liquidity management. The table also shows that the correlation coefficient between short-term loans and long-term investments is 0.151 (***), and although this value is small, it still suggests that short-term loans have a somewhat supportive effect on long-term investments. Firms may utilize short-term financing to support their long-term investment plans, especially in an uncertain financing environment.

The relationship between corporate leverage ratios and other financial indicators also provides important contextual information. Corporate leverage shows a significant negative correlation with the variable assets ratio, current ratio and return on assets. This negative correlation may reflect the challenges of financial risk management for highly leveraged firms, especially in terms of liquidity and profitability.

Variance inflation factor (VIF) is a measure of correlation between independent variables, and in general, when the VIF value is greater than 10, it is considered that there is serious multicollinearity. According to the data in Table 4, the VIF values of all variables are lower than 10, with the highest being 4.091 (return on assets), which indicates that the correlation between the independent variables is relatively low. In addition, the closer the 1/VIF value is to 1, the lesser the problem of multicollinearity. In the table, most of the variables have 1/VIF values between 0.2 and 0.5, showing a moderate linear relationship. The average VIF is 3.072, which is significantly lower than 10, which further supports that the model does not have a serious multicollinearity problem. Therefore, the correlation between the independent variables of the model does not significantly affect the results of the regression analysis

**Table 4:** Specific calculation instructions for each variable.

Variable	VIF	1/VIF
TANG	3.768	0.155
ROA	4.091	0.257
SIr	3.168	0.200
ROE	3.730	0.385
LIr	2.718	0.350
CURR	1.656	0.494
SFLI	1.819	0.710
Growth	1.019	1.044
HLD	0.983	1.071
Mean VIF	3.072	

### 4.3 Regression analysis

To enhance the understanding of the factors affecting corporate leverage, this research examines panel data through a fixed-effects regression analysis, emphasizing the impact of various financial metrics on corporate leverage. Particular attention is given to the tangible asset ratio and return on assets. The basic regression outcomes presented in Table 5 indicate that in the fixed-effects model, corporate leverage, along with short-term and long-term leverage, is both significantly and inversely influenced by the tangible asset ratio and return on assets. The regression coefficient of tangible asset ratio is −0.450 (p < 0.001), which indicates that firms are significantly less leveraged when they have more tangible assets. The results of the study show that firms’ asset structure plays a central role in financing decisions, and that high gearing tends to prompt firms to adopt more prudent financing strategies in order to reduce financial risks. Firms with rich tangible assets have a greater advantage in the credit market and can more easily obtain financing at low cost, thus optimizing their capital structure.

**Table 5:** Specific calculation instructions for each variable.

	(1)	(2)	(3)
	Lr	SIr	LIr
SFLI	0.022***	0.031***	−0.002
	(0.003)	(0.002)	(0.002)
Growth	−0.005***	−0.002	0.000
	(0.001)	(0.003)	(0.001)
TANG	−0.450***	−0.315***	−0.253***
	(0.005)	(0.005)	(0.004)
CURR	−0.004***	−0.021***	−0.007***
	(0.000)	(0.000)	(0.000)
set return rate	−0.078***	−0.069***	−0.210***
	(0.016)	(0.010)	(0.012)
ROE	−0.020***	−0.028***	−0.057***
	(0.004)	(0.006)	(0.005)
HLD	−0.060***	−0.220***	−0.205***
	(0.010)	(0.013)	(0.010)
_cons	0.868***	0.784***	0.153***
	(0.019)	(0.019)	(0.010)
N	23742	23742	23742

In addition, there is a significant negative correlation between return on assets and leverage with a regression coefficient of −0.078 (p < 0.001), which further confirms the effect of profitability on leverage. Firms with high profitability are more capable of relying on internal funds for reinvestment and reducing their dependence on external debt, thus reducing leverage. The profitability of an enterprise not only concerns its cash flow position, but also directly affects the trust of investors and creditors, which in turn has a profound impact on financing conditions. Therefore, while improving profitability, enterprises should also pay attention to improving the efficiency of capital utilization to ensure sustainable development.

The regression analysis related to the liquidity ratio reveals an important trend with a coefficient of −0.004 that is highly statistically significant (p < 0.001), especially when short-term leverage is taken into account, where the coefficient widens to −0.021. Firms that are illiquid are likely to experience more pressing short-term financing challenges, as lower liquidity ratios limit the firm’s ability to access funds in emergencies and may in turn destabilize its operations. ability in emergencies, which in turn may shake its operational stability. Therefore, liquidity management is an integral part of a firm’s financial strategy, and firms need to maintain an appropriate level of liquidity in order to flexibly respond to contingencies.

On the other hand, the regression analysis of return on net assets and the shareholding ratio of major shareholders also brings some warnings. The coefficient of majority shareholding is −0.060, which is also highly statistically significant (p < 0.001), especially in the analysis of short-term leverage, where the coefficient increases significantly to −0.220. When the shareholder structure is overly concentrated, the financial decision-making within the firm may be plagued by conflicts of interest, which in turn increases financial risk. Therefore, in order to safeguard the fairness and transparency of decision-making, firms should seek to construct a more balanced shareholder structure.

### 4.4 Robustness analysis

#### 4.4.1 Dependent variable substitution analysis.

The long-term debt-to-capital ratio visualizes the proportion of a company’s non-current liabilities in long-term capital and is a key indicator of the degree of leverage. The leverage ratio, on the other hand, usually referred to as the gearing ratio, is an important tool for assessing a company’s financial soundness and risk level, as is the long-term capital debt ratio.

According to the data in Table 6, the effect of short-term loans for long-term investments on corporate leverage is significant. Specifically, the coefficient of the effect of short-term loans on firms’ leverage is 0.0291 (p < 0.001), indicating that an increase in short-term financing significantly increases firms’ overall leverage. This result is consistent with our analysis of the long-term debt-to-capital ratio, suggesting that short-term loans contribute to a certain extent to increase the debt ratio of firms, especially when firms need to invest in long-term projects.

**Table 6:** Specific calculation instructions for each variable.

	(1)	(2)	(3)
	Lr	Short leverage ratio	LIr
SFLI	0.0291***	0.015***	0.193***
	(0.002)	(0.005)	(0.026)
Growth	−0.005***	−0.008***	−0.061***
	(0.001)	(0.003)	(0.014)
TANG	0.032***	0.036***	0.034***
	(0.003)	(0.009)	(0.039)
CURR	0.029***	0.031***	0.027***
	0.000	(0.001)	(0.005)
ROA	0.022***	0.034***	0.034***
	(0.009)	(0.023)	(0.154)
ROE	0.021***	0.026***	0.023***
	(0.004)	(0.008)	(0.062)
HLD	0.024***	0.023***	0.033***
	(0.008)	(0.019)	(0.101)
_cons	0.032***	0.034***	0.027***
	(0.022)	(0.049)	(0.177)
N	23742	23742	23742

Further examination of other factors reveals that a firm’s growth potential is inversely related to leverage, particularly in the context of long-term leverage (coefficient of −0.061, p < 0.001). This indicates that enhancing a firm’s growth potential could help mitigate leverage risk. Additionally, factors such as the ratio of tangible assets, current ratio, asset returns, and returns on shareholder equity significantly influence leverage, highlighting the intricate nature of a firm’s financial structure.

The impact coefficient of the tangible assets ratio is 0.032 (p < 0.001), indicating that firms with more tangible assets are better able to manage their leverage risk and therefore prefer to use their own funds for investment. The results of the liquidity ratio show a positive correlation with leverage, especially in the short-term leverage analysis (coefficient of 0.029), which suggests that firms with more liquidity have an advantage in financing and can effectively reduce financial risk.

By replacing the dependent variable, the study verifies the effect of short-term loans for long-term investments on the leverage ratio of firms and its robustness in the context of long-term debt-to-capital ratio.

#### 4.4.2 Lag the explanatory variable by one and two periods.

The study analyzes the lagged effect of short-term loans for long-term investment, with special attention to its impact on corporate leverage. According to the data in Table 7, the lagged one cycle of short-term loans (L. SFLI) has a significant effect on corporate leverage with a coefficient of 0.036 (p < 0.001). This suggests that the use of short-term loans in the past cycle still has a positive impact on current leverage, reflecting the financing inertia that firms may have developed in their long-term investment decisions. This persistent effect suggests that after the initial use of short-term loans, firms tend to continue to rely on that type of financing in their subsequent financial decisions, thus pushing up the leverage ratio.

**Table 7:** Specific calculation instructions for each variable.

	(1)	(2)	(3)
	Lr	SIr	LIr
SFLI	0.026***	0.042***	0.021***
	(0.002)	(0.002)	(0.003)
Growth	−0.008***	−0.002***	−0.009***
	(0.001)	(0.001)	(0.001)
TANG	−0.524***	−0.510***	−0.676***
	(0.003)	(0.003)	(0.004)
CURR	−0.007***	−0.006***	−0.008***
	0.000	0.000	0.000
ROA	−0.082***	−0.058***	−0.075***
	(0.009)	(0.010)	(0.009)
ROE	−0.021***	−0.028***	−0.026***
	(0.004)	(0.003)	(0.005)
HLD	−0.048***	−0.044***	−0.059***
	(0.012)	(0.013)	(0.015)
L. SFLI		0.036***	
		(0.001)	
L2. SFLI			0.034
			−0.002
_cons	0.836***	0.841***	0.862***
	(0.002)	(0.002)	(0.002)
N	23742	19501	17334

On the other hand, the short-term loan variable lagged two periods does not show a significant effect (coefficient of −0.002), suggesting that its effect on firms’ leverage weakens over time. Firms’ financing strategy adjustments in long-term investments may prefer alternative financing methods to reduce leverage risk over time. In addition, variables such as firms’ growth potential, current ratio, and return on assets also exhibit negative correlations with leverage, further emphasizing the importance of considering the time factor when formulating financing strategies.

### 4.5 Heterogeneity analysis

#### 4.5.1 Impact analysis of different variables.

Table 8 examines that the effect of short-term loans on firm leverage shows significant heterogeneity across indicators. Specifically, the coefficient of short-term loans for long-term investments is positive across all leverage indicators, indicating the enhancing effect of short-term loans on firm leverage. In particular, short-term leverage has the highest impact (0.038***), while long-term leverage has a slightly lower impact (0.0325***). Firms’ growth potential is negatively correlated with the leverage ratio, indicating that the higher the firm’s growth potential, the lower its leverage ratio. In particular, the effect is most significant in the long-term leverage ratio (−0.006***). The relationship between the tangible assets ratio and the leverage ratio is also evident, with negative coefficients indicating that the higher the tangible assets ratio, the lower the leverage of the firm, with particularly significant effects in the short- and long-term leverage ratios (−0.866*** and −0.699***).

**Table 8:** Specific calculation instructions for each variable.

	(1)	(2)	(3)
	Lr	SIr	LIr
SFLI	0.038***	0.014***	0.0325***
	(0.002)	(0.003)	(0.002)
Growth	−0.004***	−0.002***	−0.006***
	(0.001)	(0.003)	(0.002)
TANG	−0.866***	−0.889***	−0.699***
	(0.004)	(0.005)	(0.004)
CURR	−0.007***	−0.014***	−0.004***
	0	−0.00119	0
ROA	−0.082***	−0.058***	−0.068***
	(0.011)	(0.022)	(0.018)
ROE	−0.036***	−0.028***	−0.037***
	(0.004)	(0.006)	(0.007)
HLD	−0.062***	−0.07	−0.058***
	(0.011)	(0.011)	(0.013)
_cons	0.708***	0.675***	0.667***
	(0.018)	(0.016)	(0.023)
N	23742	10039	11629

#### 4.5.2 Impact of industry and time grouping.

The study further analyzes the differences in the impact of short-term loans across industries and time periods. The results in Table 9 show that in the analysis grouped by time, the impact of short-term loans is significant before 2015, especially in the short-term leverage ratio (0.028***). In contrast, in the data for 2015 and later, although the coefficient of short-term loans increased (0.032***), there is a tendency to weaken in terms of statistical significance, and some of the indicators even have negative values (−0.002). In the analysis by industry grouping, the effect of short-term loans in the manufacturing sector is the most significant (0.035***), indicating that the manufacturing sector is more dependent on short-term loans. The results for service and other industries also show a positive correlation, but the impact is relatively small (0.025** for service and 0.029*** for other industries). This suggests that there is a significant difference in the response of different industries to short-term loans and that the manufacturing sector may be more dependent on short-term loans in its financing structure.

**Table 9:** Time- and Industry-Varying Impact of Short-Term Loans for Long-Term Investment Effects on Firm Leverage Ratio.

	Lr (1)	SIr (2)	SIr (3)
Group by time			
Pre-2015	0.028***	0.017***	0.033***
	(0.003)	(0.004)	(0.003)
2015 and beyond	0.032***	0.021***	0.037***
	−0.002	−0.003	−0.002
Grouping by Industry			
manufacture	0.035***	0.022***	0.040***
	(0.004)	(0.005)	(0.004)
services sector	0.025**	0.016**	0.030**
	(0.005)	(0.006)	(0.005)
Other industries	0.029***	0.018***	0.034***
	(0.003)	(0.004)	(0.003)

#### 4.5.3 Impact analysis in the Context of COVID-19.

During the COVID-19 pandemic, firms faced unprecedented liquidity crisis and financial uncertainty, so the strategy of short-term loans for long-term investment became an important choice for firms to cope with the challenge. According to the results of the empirical analysis in Table 10, the coefficient of short-term loans for long-term investments is 0.030***, which indicates a significant positive relationship between this strategy and firms’ leverage ratios during this particular period. This result highlights the key role of short-term financing restructuring in optimizing firms’ capital structure, especially in the case of liquidity constraints, and firms should prioritize the enhancement of financial stability through this type of financing strategy.

**Table 10:** The effect of short-term loans on the mechanism of corporate leverage ratios.

	Lr (1)	SIr (2)	LIr (3)
SFLI	0.030***	0.019***	0.035***
	(0.004)	(0.046)	(0.004)
Growth	−0.006***	−0.006***	−0.007***
	(0.008)	(0.034)	(0.001)
TANG	−0.677***	−0.723***	−0.648***
	(0.005)	(0.006)	(0.005)
CURR	−0.007***	−0.012***	−0.005***
	(0.010)	(0.026)	(0.008)
ROA	−0.085***	−0.076***	−0.068***
	(0.004)	(0.005)	(0.002)
ROE	−0.027***	−0.027***	−0.030***
	(0.012)	(0.003)	(0.043)
HLD	−0.060***	−0.056***	−0.066***
	(0.039)	(0.204)	0.954
_cons	0.920***	0.925***	0.749***
	(0.018)	(0.059)	(0.001)
*N*	21668	10039	11629

Further analysis shows that variables such as firm growth potential, proportion of reachable assets, current ratio and return on assets all have a significant negative impact on the leverage ratio. For example, the coefficient of firm’s growth potential is −0.006***, indicating that when the firm’s growth potential increases, its leverage level tends to decrease, reflecting the firm’s preference for controlling its liabilities in order to achieve sustainable development. In addition, the significant negative effect of the ratio of reachable assets (with a coefficient of −0.677***) suggests that a higher asset base enables firms to finance themselves more efficiently and reduce their reliance on external liabilities. Clearly, the COVID-19 epidemic has had a profound impact on corporate financing strategies. When formulating financing strategies, enterprises must comprehensively consider all financial indicators and optimize their capital structure so as to enhance their overall financial health.

## 5. Conclusion and recommendations

In the study of the relationship between corporate financing strategies and financial stability, we pay special attention to the impact of the specific behavior of using short-term loans for long-term investment on corporate leverage. Through systematic data analysis and empirical research, we draw the following conclusions. The study shows that the use of short-term loans has a significant effect on corporate leverage. Although in the short term, this financing tool can effectively respond to the enterprise’s capital needs, in the long term, frequent or excessive reliance on short-term loans to support long-term investment will lead to increased instability in the enterprise’s financial structure, which will significantly increase the enterprise’s debt service risk. In addition, the study shows that there is a significant difference between different types of enterprises in the impact of short-term loans on leverage. State-owned enterprises (SOEs) usually prefer long-term loans because they enjoy lower financing costs and stronger access to capital. As a result, the impact of short-term loans on the leverage ratio of state-owned enterprises is relatively small, while non-state-owned enterprises rely more on short-term loans and thus their leverage ratio is more significantly affected.

The results of the study provide an important reference for enterprise financing decisions. By clarifying the potential risk of short-term loans on long-term investment, corporate managers can choose financing methods more prudently and optimize the capital structure so as to improve financial stability. Meanwhile, this study enriches the related literature on the theory of corporate financing and leverage. By analyzing the relationship between short-term loans and corporate leverage, the study provides a new perspective for understanding the behavioral differences between different types of firms in their financing decisions and emphasizes the importance of financing structure in corporate financial management.

However, the sample limitation of this study also needs to be emphasized. The fact that the study focuses only on Chinese listed firms and that Chinese firms generally have a financing structure with a high proportion of short-term loans may limit the generalizability of the findings. Therefore, future studies may consider expanding the sample to other countries and regions to verify the generalizability of the impact of short-term loans on corporate leverage. A more comprehensive understanding of the mechanism of the impact of short-term loans on corporate leverage in different economic environments can be achieved through a comparative analysis of different countries and regions.

## Supporting information

S1 Table The financial data of listed companies. (XLSX)
